# Autonomous Mission of Multi-UAV for Optimal Area Coverage

**DOI:** 10.3390/s21072482

**Published:** 2021-04-02

**Authors:** Youkyung Hong, Sunggoo Jung, Suseong Kim, Jihun Cha

**Affiliations:** Electronics and Telecommunications Research Institute (ETRI), Daejeon 34129, Korea; youkyh1@etri.re.kr (Y.H.); suseongkim@etri.re.kr (S.K.); jihun@etri.re.kr (J.C.)

**Keywords:** unmanned aerial vehicle, mission planning, area coverage, task assignment, mixed integer linear programming, path planning

## Abstract

This study proposes an entire hardware and software architecture from operator input to motor command for the autonomous area coverage mission using multiple unmanned aerial vehicles. Despite the rapid growth of commercial drone services, there are many limitations on operations, such as a low decision-making autonomy and the need for experienced operators to intervene in the whole process. For performing the area coverage mission more efficiently and autonomously, this study newly designs an optimization problem that allocates waypoints created to cover that area to unmanned aerial vehicles. With an optimized list of waypoints, unmanned aerial vehicles can fill the given areas with their footprints in a minimal amount of time and do not overlap each other during the mission. In addition, this study performs both various simulations for quantitative analysis and an outdoor experiment through real hardware implementation in order to verify the performance sufficiently. The methodologies developed in this study could be applied to endless applications using unmanned aerial vehicles equipped with mission-specific sensors.

## 1. Introduction

Unmanned Aerial Vehicles (UAVs), commonly known as drones, will replace most human labor, especially in dangerous places and tedious tasks. The world’s largest drone companies (e.g., DJI, Parrot, and 3DR) have already launched commercial drone service in various fields such as agriculture, mapping, and infrastructure inspection. Unfortunately, at the current level of service, there is a limit to the number of drones to be performed simultaneously because a skilled human operator must intervene in the entire process of the operation. However, to overcome the small payload capacity and short operating range of a single drone, it is essential to deploy multiple drones at industrial sites and increase autonomy. Researchers also have increased interest in the autonomous operation of drones using artificial intelligence, but it is still difficult to apply them to a single drone. For these reasons, research on autonomous missions using multiple drones is still in its early stages.

Otto et al. [1] classified the autonomous missions that can be performed by UAVs into area coverage [2], search [3], routing [4,5], data collection, and communication relay: the area coverage mission applies to building inspection and pesticide spraying; the search mission applies to rescue service and wildfire suppression; the routing mission applies to transport and parcel delivery. The main difference between the area coverage and search missions is whether the environment in which the mission is performed is previously known or not. The area coverage mission first defines a finite area and then makes UAVs thoroughly monitor that area with equipped sensors. On the other hand, the search mission aims to detect particular targets in an unknown environment. Therefore, a different strategy with the coverage mission is needed to accurately estimate the target location and expand the search area in a minimal amount of time. Note that the methodologies proposed for the area coverage mission can be the simplest solution for the search mission. When the area coverage method applies to the search mission, UAVs gradually scan the entire area regardless of time and eventually encounter the target. For that reason, this study takes the area coverage mission as a starting point. In the following, we will review recent research related to the area coverage mission.

Most of the previous research carrying out the coverage mission has focused on generating a coverage pattern for exploring the given area with a single drone. Typical geometric patterns to explore the given area are divided into the back-and-forth movement [2], spiral pattern [6], and grid-based method [7]. Among several coverage patterns, the back-and-forth movement can be considered the most energy-efficient [1]. The reason is that when the UAV changes its flight direction by making a sharp turn, it needs to slow down, rotate, and then speed up again. Thus, as the number of turning maneuvers increases, the UAV consumes more energy [8].

On the other hand, only a few studies have performed the area coverage mission using multiple vehicles. Most of them have adopted a two-step procedure consisting of (i) area partitioning and (ii) subarea assignment. Peterson et al. developed a multi-robot system of UAV and UGV to localize radioactive materials by covering a given area [9]. Barrientos et al. presented a multi-UAV system for aerial imaging applied to precision agriculture and conducted extensive field tests [10]. In their works, after the preprocessing of dividing a given area into several smaller subareas and assigning the divided subareas to unmanned vehicles, each unmanned vehicle performs coverage path planning to explore the allocated subareas. However, a considerable effort is required to decompose the given area into subareas reasonably and then effectively assigns the subarea to each UAV, considering the capability of UAVs. In contrast, Avellar et al. proposed a novel approach that allows multiple UAVs to achieve cooperative area coverage by increasing the vehicle’s decision-making autonomy without dividing the area in advance [2]. To be more specific, a given area could be transformed into a list of waypoints by applying the back-and-forth movement. As UAVs have to visit the list of waypoints, the area coverage mission can eventually be modeled as the multiple Traveling Salesman Problem (TSP). Aevellar et al. used Mixed Integer Linear Programming (MILP) to solve the TSP. Various optimization methods such as metaheuristics [11] and hybrid methods [12] allow us to calculate the optimal solutions of the TSP.

It is also important to configure the entire hardware and software system from operator input to motor command. However, relatively few studies have been devoted to the construction of the entire mission execution framework. Valente et al. proposed taking aerial images and building a map through image stitching for precision agriculture [13]. In their system, the mission area is sampled with a grid of constant size, and then a path is generated to perform full coverage with the minimum number of turns without revisiting the grid. However, their work was limited in that only one UAV participated in the mission, and that area information to perform coverage was provided to the UAV in advance. Garzon et al. presented a multi-robot system including software, hardware, and communication architecture for a signal search mission [14]. However, their system applied to Unmanned Ground Vehicles (UGVs), not UAVs, and thus focused on comparing two different approaches to coverage path planning techniques (i.e., the back-and-forth movement and the spiral pattern). Besides, few studies have conducted field tests to verify the performance of the mission system. Nedjati et al. presented a new multi-tour coverage for a post-earthquake response system that collects images at the earthquake site and builds a map to extract useful information [15]. Yao et al. proposed inspecting urban buildings by assigning buildings to UAVs and generating optimal spherical coverage patterns around the buildings [16]. However, both previous studies presented only the results of numerical simulations without performing experiments using real hardware. Acevedo et al. studied a more practical coverage algorithm in which subareas are assigned to heterogeneous aerial robots, taking into account their various sensing and motion capabilities [17]. Mansouri et al. developed a more sophisticated coverage method that considers the camera movement and acquired the stitched image by collecting image streams during the coverage mission [18]. However, these previous studies are still unfortunate as indoor flight tests have been conducted in limited space because most practical applications of the area coverage mission run outdoors. In other words, indoor experiments cannot be sufficient validation because there is no wind and no GPS errors (thanks to motion capture system), which might be critical and challenging issues in a real implementation.

The primary purpose of this study is to provide the entire mission system configuration for the area coverage mission using multiple UAVs and to design the optimization problem for task assignment. More exactly, the autonomous area coverage mission begins when the operator enters parameters to define the mission through the Ground Control Station (GCS). This study designs an optimization problem to assign optimal waypoint lists to UAVs based on MILP. The optimization problem takes into account the back-and-forth movement and is intended to completely fill the given area with the footprint of multiple UAVs. When each UAV receives an optimal list of waypoints, it takes off from the ground and visits a series of waypoints in order. During the mission, the waypoint status is monitored in the proposed task management algorithm. The entire mission ends with all UAVs flying through this process and then landing. Accordingly, the following assumptions are considered in this study. First, we assume that the area covered by multiple UAVs exists in a two-dimensional space and is always convex. Second, the given area is small enough to allow the UAV to complete the mission with one full charge, and therefore we do not consider returning to the depot to recharge the battery. Third, it is assumed that all UAVs participating in the mission have the same capability (i.e., the maximum travel distance). Forth, we consider the centralized task assignment scheme performed on GCS, so the assignment results are unilaterally transferred once to UAVs waiting on the ground. In addition, we assume that the communication range and the amount of data required for the communication between the UAV and GCS is unlimited. Lastly, we envision that the mission environment is static and clean without obstacles. It means that there are no significant problems with UAVs following the assigned waypoints, so no additional strategies are required to respond to environments with static obstacles or dynamic environments.

The contributions of this study are threefold. First, this study presents a mission execution framework ranging from operator input to motor command to perform the area coverage mission. Second, by comparison with the motivational research [2], this study expands to multiple areas and lightens the computational complexity of optimization. Third, this study addresses various simulations and flight tests in outdoor environments to verify the proposed system’s performance. To the best of our knowledge, we provide the first multiple areas optimal coverage with multiple drones verified through outdoor experiments.

The rest of this study is organized as follows. Section 2 defines each subsystem constituting the entire system and the interactions between subsystems. Section 3 explains the graph building process for the task assignment subsystem in detail and provides the MILP formulation, including objective function and constraints. In Section 4, the proposed system’s performance is verified through various simulations performing single area coverage missions with different types of polygons and different numbers of UAVs. Section 5 presents the results of the outdoor experiment in which two hexacopters cover multiple areas designated by the operator. Section 6 describes this study’s conclusions and discusses possible directions for future research.

## 2. Problem Description

**Figure 1:**
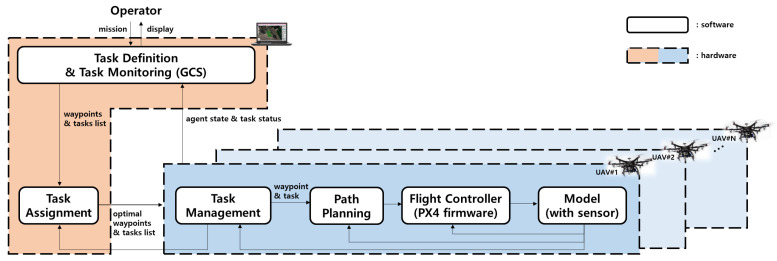
System architecture.

Figure 1 shows the hardware and software architecture of the entire mission system from operator input to motor command. As shown in Figure 1, the primary components of the hardware are multiple UAV platforms and one laptop computer that acts as a ground-based GCS hardware system. In terms of the software, the entire mission system consists of five subsystems; GCS software for task definition and task monitoring, task assignment, task management, path planning, and flight control subsystems. All of these subsystems work under the Robot Operating System (ROS) framework [19]. Note that GCS software and task assignment subsystems work on the laptop computer. On the other hand, task management, path planning, and flight control subsystems operate on an on-board computer mounted on each UAV platform. The subsections below describe each subsystem’s operations; however, the description of the flight control subsystem is omitted because the PX4 firmware [20], which is an open-source autopilot software, is used as the flight control subsystem.

### 2.1. GCS Software

In this study, the GCS software is developed based on QGroundControl [21], which is an open-source GCS working with various vehicle types supported by the PX4 firmware. In the original QGroundControl, the operator enters several parameters to define the survey, such as a polygonal area, the angle between waypoints, the spacing between waypoints, and the altitude to perform the mission. Then, QGroundControl creates a set of waypoints based on the operator inputs. Note that the waypoints generated by the original QGroundControl are (i) highly operator dependent, (ii) not optimized, and (iii) not for multiple UAVs.

The following four features are newly added to the original QGroundControl for the optimal area coverage mission using multiple UAVs aimed in this study. First, QGroundControl is modified to be able to publish and subscribe to ROS messages. For QGroundControl to work in the ROS environment, a WebSocket connection is selected to design the client structure. The rosbridge interface [22] is implemented to communicate with other subsystems. Therefore, the operator inputs through QGroundControl are transferred to other subsystems as ROS messages. In addition, the results calculated by other subsystems are sent as ROS messages so that the operator can monitor the progress of the mission. Second, QGroundControl is expanded to perform missions using multiple UAVs. To do this, the operator needs to enter two additional inputs: the number of UAVs and the origin of the inertial coordinate. In addition, the position of each UAV in the GNSS coordinate is sent to the modified QGroundControl. After that, the relative coordinates between the inertial coordinate and the body-fixed coordinate of each UAV are defined, and the coordinate transformations are performed. For that reason, despite the operator defining the mission areas in the inertial coordinate, each UAV can transform the waypoints assigned to it in its body-fixed coordinate. Third, even if the operator does not specify the direction of the back-and-forth movement (i.e., the angle between waypoints), the proposed task assignment subsystem determines the optimal direction to cover the polygonal area. Therefore, although the operator enters fewer parameters to define the mission than the original QGroundControl, the operator can be provided with optimal mission planning. Namely, the modified QGroundControl in this study attempts to determine the optimal waypoints for multiple UAVs rather than simply flying along waypoints entered by the operator.

In summary, the primary operations of GCS software are (i) task definition determining which waypoints UAVs should visit to perform the mission and (ii) task monitoring to see the progress of the mission. The GCS software interacts with the operator and other subsystems at 1 Hz as follows. The inputs entered by the operator are the vertices of the polygonal areas, the mission altitude, the number of UAV, the spacing of waypoints, and the origin of the inertial coordinate. In addition, the GCS software needs the longitude and latitude of each UAV obtained from the GPS sensor mounted on each UAV. As the outputs for the operator, the GCS software displays the list of optimal waypoints determined by the task assignment subsystem and the status of each waypoint provided by the task management subsystem on the map. The outputs to other subsystems are the operator inputs and the relative coordinates between the inertial and the body-fixed coordinates.

### 2.2. Task Assignment

The primary function of the task assignment subsystem is to allocate waypoints to each UAV in order to perform the mission given by the operator optimally. The inputs required for the task assignment subsystem are the vertices of the polygonal areas, the number of UAV, and the initial position of UAV in the inertial coordinate. The outputs of the task assignment subsystem are the set of waypoints assigned to each UAV and are provided at the end of solving the optimization problem. As has been mentioned in the previous section, task reassignment is not considered in this study. However, if task reassignment is required, this subsystem needs additional inputs from the task management subsystem to solve the task reassignment problem. For example, the task reassignment problem requires additional information, such as which UAV is failed to reach the given waypoint and the residual list of waypoints. The task assignment subsystem will be discussed in detail in Section 3.

### 2.3. Task Management

**Figure 2:**
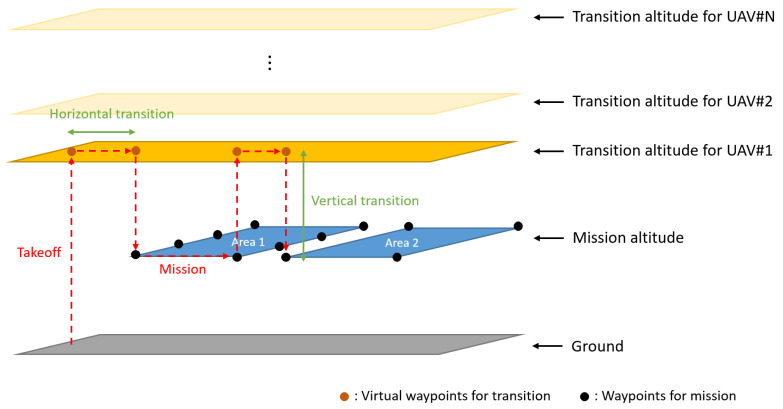
A concept of transition altitude.

Task management identifies the target waypoint that a UAV should currently head to in the list of waypoints and checks whether the UAV reaches the target waypoint or not. The optimal waypoint list is subscribed from the task assignment subsystem. Because this study considers two-dimensional area coverage, the altitude of the waypoints is equal to the mission altitude defined by the operator. It is necessary to prevent collisions with other UAVs in the process of starting from the depot and moving to the mission area, moving between different polygon areas, and returning to the depot after completing the mission. Therefore, in this study, the transition altitude concept is newly introduced, where each UAV is assigned a unique transition altitude. Inside the polygonal area, a lateral safety separation is ensured due to the spacing of the waypoints. Outside of the polygonal area, a longitudinal safety separation is possible thanks to the transition altitude. In other words, as shown in Figure 2, we consider virtual waypoints in addition to the waypoints for the coverage mission to ensure safe separation between UAVs.

When a UAV enters within a certain radius of the target waypoint, it is considered that the UAV reached the target waypoint. Although the task reassignment problem is beyond this study’s scope, the following strategy can be envisioned when a UAV fails to reach the target waypoint. First of all, the task management subsystem can retry the same task (i.e., visiting the target waypoint) a certain number of times. Nevertheless, if the retrial fails, the task management subsystem decides that the remaining mission should be reassigned and transfers the decision with additional information required to solve the reassignment problem to the task assignment subsystem.

In short, the inputs of the task management subsystem are the optimal waypoint list assigned for each UAV. The outputs of the task management subsystem are the status of each waypoint and information related to the task reassignment problem. Note that the task management subsystem operates at 5 Hz.

### 2.4. Path Planning

The role of path planning is to generate a guidance command at 50 Hz that allows a UAV to navigate to the target waypoint designated by the task management subsystem. In this study, linear velocity command $V_{cmd}\in R^{3}$ to the X-Y-Z axis of the body-fixed coordinate is considered as the guidance command to be transferred to the flight control subsystem. Therefore, the position error between the target waypoint $P_{des}\in R^{3}$ and the UAV’s position $P\in R^{3}$ is defined in the body-fixed coordinate. In order to make the position error converge to zero, the linear velocity command can be derived based on a proportional controller as follows:

(1) $$V_{cmd}=-K\left(P-P_{des}\right)$$

where *K* denotes the proportional gain matrix. In addition, the predefined maximum speed $V_{max}$ is set for stable flight. The velocity command $V_{cmd}$ is limited to ensure proper waypoint followings without exceeding the maximum speed $V_{max}$ as follows.

(2) $$|V_{cmd}|\leq V_{max}$$

## 3. Methodologies for Task Assignment

This section describes the procedures and methods used in the task assignment subsystem in detail. Firstly, a graph consisting of nodes and edges is constructed as a two-dimensional square matrix. Under the constructed graph, an optimization problem is designed based on MILP to determine the optimal pair between UAVs and waypoints.

### 3.1. Graph Building

When the vertices of the polygonal areas, the number of UAV, the initial position of UAV in the inertial coordinate, and the spacing between waypoints are provided from the GCS software, the task assignment subsystem’s first operation is to build a graph consisting of nodes and edges. As the simplest way to fill the finite two-dimensional area with each UAV’s footprint, the area to be covered can be gridded with the footprint size of each UAV. Therefore, the area to be covered can be expressed as a graph composed of nodes representing the center of the footprint and edges connecting nodes. However, as mentioned previously, this study considers the back-and-forth movement, not the grid-based method. The following describes the detailed procedure of building a graph to cover the given areas with the back-and-forth movement.

**Figure 3:**
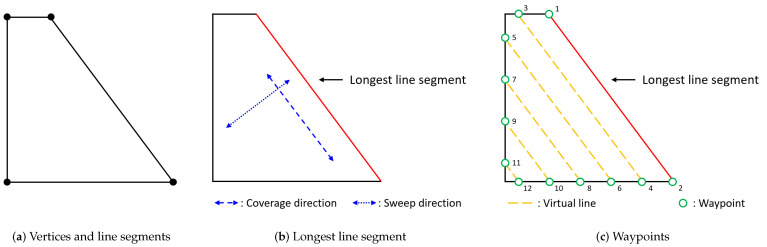
Graph building procedures.

First, the node consists of (i) each UAV’s initial launch position (called a depot) and (ii) the waypoints that each UAV should visit. The waypoints can be generated as follows. When the polygonal area is given as a sequence of vertices, each line segment connecting two vertices can be determined as the polygon boundaries. In other words, by drawing line segments connecting two adjacent vertices in a clockwise (or counter-clockwise) direction, a set of line segments whose number is equal to the number of vertices can be determined. For example, in the tetragon shown in Figure 3a, the polygon boundaries are connected to four line segments. The longest line segment among the boundaries is determined and is regarded as the coverage direction (perpendicular to the sweep direction) [2]. For example, the relationship between the longest line segment, the coverage direction, and the sweep direction is illustrated in Figure 3b. Virtual lines are generated parallel to the coverage direction. The intersections between the virtual lines and the boundaries can be considered as the waypoints. For example, 12 intersections can be determined by placing the virtual lines parallel to the longest line segment at intervals of 1 m in a polygonal area, as shown in Figure 3c. These 12 intersections are regarded as the waypoints that UAVs should visit.

Secondly, the edge can be determined as follows. The graph can be considered as a two-dimensional square matrix of which size is equal to the number of nodes. The element (i,j) of the two-dimensional square matrix corresponds to the edge connecting the *i*-th waypoint and the *j*-th waypoint. In general, the value of the element (i,j) (i.e., the cost of the edge) is determined by the distance between nodes. However, in the following three cases, a different value is used as the edge cost instead of the distance between nodes for particular purposes. The first case is that the value of the diagonal element (i,j) when i=j is set to an arbitrarily large number. This strategy has been commonly used to prevent tours from node *i* to node *i* in the TSP. The second case is to prevent a UAV from visiting other UAV’s initial launch position. To this end, if node *i* and node *j* correspond to the initial launch position of UAV, the distance between node *i* and node *j* is multiplied by a penalty greater than one. In general, when performing coverage or routing missions with multiple UAVs, only one depot was considered in most previous studies [23]. However, when considering a single depot, time separation between UAVs is essential to guarantee safe separation at the depot [24]. For this, the time to reach the node should be considered as a decision variable. As the number of decision variables increases, the computation amount required for optimization increases. Therefore, we consider a method of spatially separating each UAV, starting from a different position. The last case is to avoid overlapping of the areas to be covered by different UAVs. After a UAV makes the back-and-forth movement along a virtual line segment, it can move to a nearby waypoint. In this study, the numbering difference between waypoints is not more than two are defined as the nearby waypoints. Therefore, if node *i* and node *j* are not be considered as the nearby waypoints, the distance between node *i* and node *j* is multiplied by a penalty greater than one.

### 3.2. Decision Variables and Constraints

Let us consider that there are *N* nodes and *M* UAVs. Each node *i* and UAV *k* belong to the set of nodes N (i.e., n(N)=N) and the set of UAVs M (i.e., n(M)=M and N>M), respectively. Note that the first *M* nodes represent the depots of each UAV, and therefore the set $N_{0}$ (i.e., $n(N_{0})=N-M$) is defined to distinguish nondepot nodes [23]. Additionally, the set $N^{\prime }$ is set to $N^{\prime }=\left\{x\;|\;x\in N_{0},\;x=\mathrm{odd}\right\}$ to distinguish the waypoints on the same sides relative to the center of the virtual lines.

Three decision variables are defined to formulate the optimization problem: one binary variables $x_{ij}^{k}$, and two slack variables *s* and $u_{i}$ for ∀i,j∈N, ∀k∈M, i≠j. Note that *i* and *j* are used for the index of nodes, and *k* is used for the index of UAVs. First, $x_{ij}^{k}$ is a binary variable which becomes one when a UAV *k* is assigned to the edge connecting node *i* and node *j* and zero otherwise. Second, *s* is a continuous slack variable representing the longest travel distance among all UAVs. Lastly, $u_{i}$ is a continuous slack variable that is required for the constraint to prevent subtours in the typical TSP [2].

There are five constraints required to determine the optimal pair between *N* nodes and *M* UAVs. The first constraint is that all UAVs should visit all nodes only once except the depots. The first constraint can be represented as follows.

(3) $$\sum_{k=1}^{M}\sum_{i=1}^{N}x_{ij}^{k}=1,\;\forall j\in N_{0}$$

The second constraint indicates that if a UAV arrives at a node, then the UAV should also depart from the node to another node. It can be summarized as follows.

(4) $$\sum_{i=1}^{N}x_{ij}^{k}-\sum_{i=1}^{N}x_{ji}^{k}=0,\;\forall j\in N,\forall k\in M$$

The third constraint is required to prevent subtours in the typical TSP with the continuous slack variable $u_{i}$. This constraint can be expressed as follows.

(5) $$u_{i}-u_{j}+N\sum_{k=1}^{M}x_{ij}^{k}\leq N-1,\;\forall i,j\in N_{0},i\neq j$$

By using the fourth constraint, all UAVs are regulated to participate in the routing mission as follows.

(6) $$\sum_{j=1}^{N}x_{ij}^{k}=1,\;\forall k\in M,\forall i\in N\backslash N_{0}$$

The last constraint is necessary to make the back-and-fourth movement [2] and can be formulated as follows.

(7) $$\sum_{k=1}^{M}x_{i(i+1)}^{k}+\sum_{k=1}^{M}x_{(i+1)i}^{k}=1,\;\forall i\in N^{\prime }$$

Note that the numbering order is critical for the last constraint in Equation (7) to work correctly. In this study, the waypoints (i.e., the nodes of the set $N_{0}$) are numbered as shown in Figure 3c. Intersections created by one virtual line have numberings that are adjacent to each other. Therefore, from the center of the virtual lines, intersections on one side are odd (or even), and intersections on the other side are even (or odd).

### 3.3. MILP Formulation

The optimization problem for assigning *N* nodes to *M* UAVs using previously defined decision variables and constraints can be summarized as follows.

(8) $$\mathrm{Minimize}\;s\;+\;\frac{1}{M}\sum_{k=1}^{M}\sum_{i=1}^{N}\sum_{j=1}^{N}c_{ij}x_{ij}^{k}$$

subject to Equations (3)–(7),

(9) $$\sum_{i=1}^{N}\sum_{j=1}^{N}c_{ij}x_{ij}^{k}\leq s,\;\forall k\in M$$

The objective function is designed to minimize the maximum travel distance *s* (i.e., the first term in Equation (8)) and the average travel distance of all UAVs (i.e., the second term in Equation (8)). Note that the constant $c_{ij}$ denotes the cost of moving the edge from node *i* and to node *j* (i.e., the element (i,j) of the graph). Lastly, Equation (9) is added to define the maximum travel distance *s*.

## 4. Simulations

This section validates the performance of the proposed framework for the area coverage mission of multiple UAVs through two simulations. In the first simulation, the task assignment subsystem explained in Section 3 is applied to various polygonal areas and different number of UAVs. The second simulation evaluates the entire system described in Section 2 by assuming that an arbitrary polygonal area is given by the operator. All numerical computations were performed using a laptop computer with a 2.6 GHz Intel i7 CPU and 16 GB RAM running the Ubuntu operating system. Note that the task assignment subsystem was written in MATLAB (version R2020a; The MathWorks Inc., Natick, MA, USA) and used Gurobi solver [25], which is a standard optimization software package for MILP, to solve the optimization problem described in Section 3.3.

### 4.1. MATLAB Simulation

The first simulation aims to verify the performance of the task assignment subsystem. In other words, this simulation focuses on determining whether the task assignment subsystem appropriately solves the optimization problem. Several different configurations are considered; two to five UAVs cover a single polygonal area randomly provided from triangle to hexagon.

**Figure 4:**
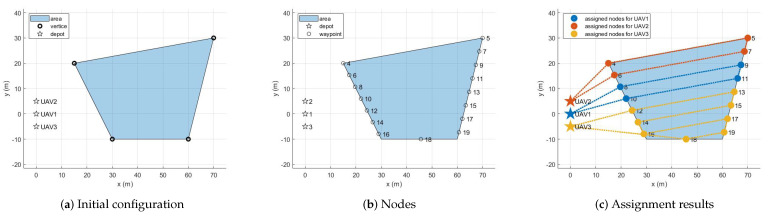
Task assignment procedures for the sample configuration.

Figure 4 shows the sample configuration in which three UAVs cover a pentagon. Suppose that the following information is provided as inputs to the task assignment subsystem: (i) the five vertices of the pentagon are given as small circles in Figure 4a, (ii) the spacing between waypoints is set to 5 m, (iii) the number of UAVs participating in coverage is three, and (iv) the UAVs are initially placed in the inertial frame as shown in the small asterisks in Figure 4a. We can use these inputs to create the graph according to the method described in Section 3.1. Figure 4b presents the nodes of the graph. The three small asterisks and 16 small circles represent the depots of each UAV and the waypoints, respectively.

To describe the input arguments of the Gurobi solver, let us reproduce the optimization problem described in Section 3 for the sample configuration. All constraints in Equations (3)–(7) and (9) can be considered matrix equations or inequalities for the solution vector, including three decision variables. Note that the solution vector has size 1103 by 1 (1083 for $x_{ij}^{k}$, 19 for $u_{i}$, and 1 for *s*). In the first constraint described in Equation (3), the linear equality constraint matrix has size 19 by 1103, where 19 is the number of nodes and 1103 is the size of the solution vector. In the second constraint explained in Equation (4), the linear equality constraint matrix has size 57 by 1103, where 57 is the product of the number of nodes and the number of UAVs (i.e., 57=19×3). The third constraint presented in Equation (5) has the linear inequality constraint matrix of which size is 240 by 1103, where 240 is equal to 240=(19−3)×(19−3)−16. The linear equality constraint matrices of the fourth constraint in Equation (6) and the fifth constraint in Equation (7) have size 3 by 1103 and size 8 by 1103, respectively. In the additional constraint described in Equation (9), the linear inequality constraint matrix has size 3 by 1103. The objective function given in Equation (8) can be expressed as the dot product between the coefficient vector and the solution vector. Lastly, we need to specify the lower and upper bounds of the solution vector *x* and specify which value is an integer. In this way, when all input arguments to the Gurobi solver are specified, the Gurobi solver provides the optimal value of decision variables as output.

Figure 4c provides the assignment results of allocating 19 nodes to three UAVs to cover the given area optimally. The computation time was only 0.80 s to solve the optimization problem. As shown in Figure 4c, the UAVs could cover the given area by implementing the back-and-forth movement without overlapping each other.

It can be seen from Figure 4c that the upper waypoints were allocated to UAV2 initially located relatively above, and the lower waypoints were allocated to UAV3 initially located relatively lower. Additionally, as Figure 4c shows, UAV3 was assigned more waypoints than UAV1 and UAV2 because the distance from the initial launch position to the first waypoint for UAV1 and UAV2 was greater than UAV3. Table 1 summarizes the number of waypoints and the sequence of waypoints assigned for each UAV, the travel distance of each UAV, and the average travel of all UAVs.

**Figure 5:**
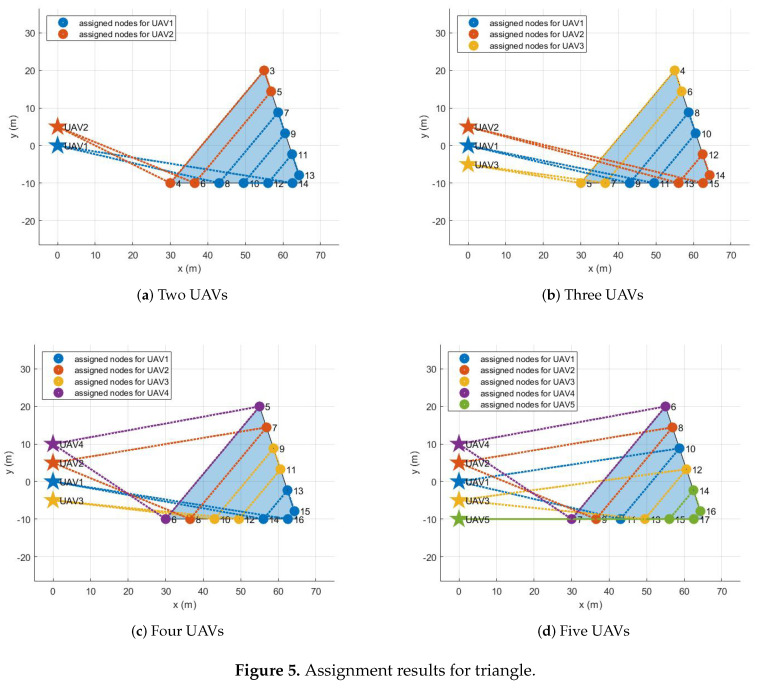
Assignment results for triangle.

**Figure 6:**
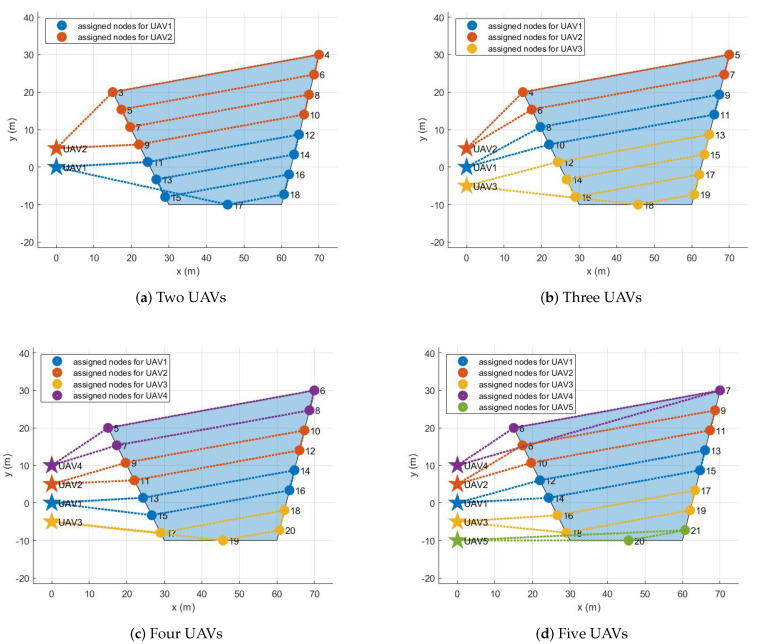
Assignment results for tetragon.

**Figure 7:**
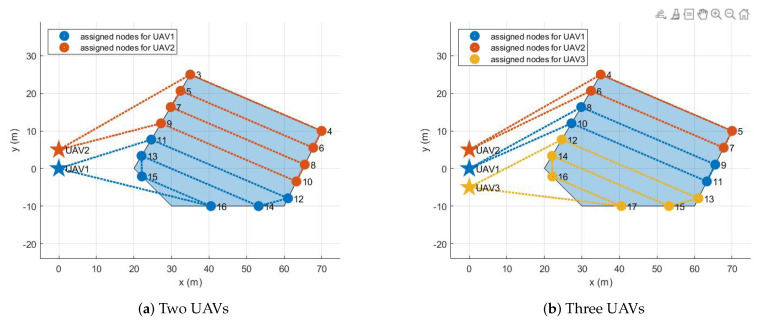

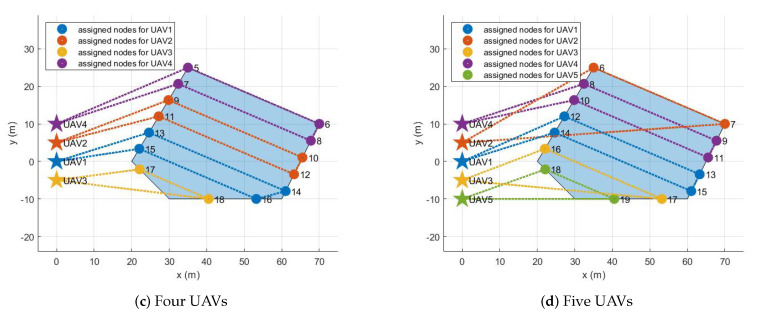
Five UAVs

**Figure 8:**
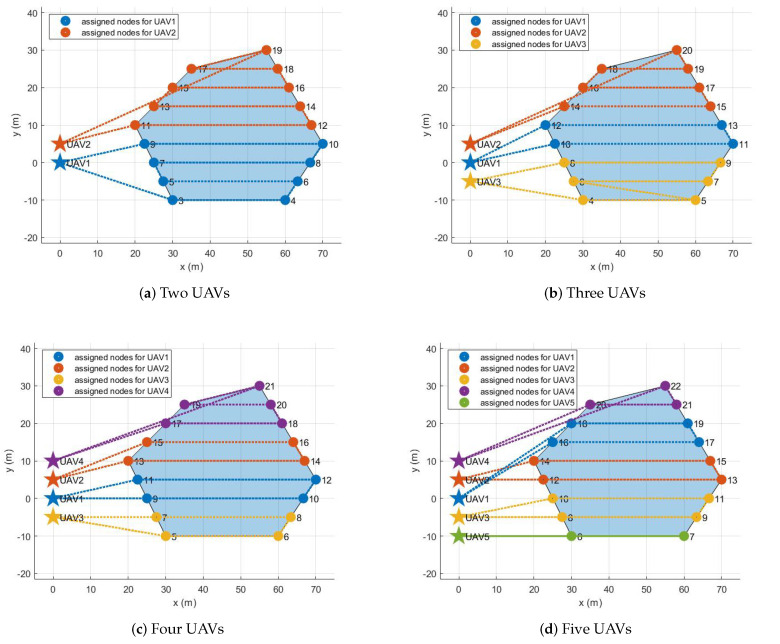
Assignment results for hexagon.

The assignment results for all configurations are described in the following. If there is one UAV, its initial position is set to (0 m, 0 m), the origin of the inertial coordinate. As the number of UAVs increases, the added UAVs are placed 5 m away along the y-axis. For example, if there are five UAVs, their initial positions are (0 m, 0 m), (0 m, 5 m), (0 m, −5 m), (0 m, 10 m), and (0 m, −10 m). Figure 5, Figure 6, Figure 7 and Figure 8 show the assignment results when the number of UAVs varies from two to five and a polygonal area from triangle to hexagon is given to cover. Table 2 summarizes the travel distance of each UAV and the average and maximum travel distance according to the assignment results. For all configurations, as we intended, waypoints were reasonably assigned to each UAV so that the polygonal area was covered by each UAV, like cutting a cake. The assignment results can also be interpreted as UAVs cooperate to minimize the objective function, the goal of all UAVs, in Equation (8) while satisfying the constraints in Equations (3)–(7) and (9). Note that, to describe the results on a limited page, we set the number of UAVs to a maximum of five and polygons to a maximum of a hexagon. However, the proposed task assignment can operate for more UAVs and more complex polygon.

### 4.2. MATLAB and Gazebo Cosimulation

The purpose of the second simulation is to evaluate the entire system performing the coverage mission as final verification before the actual flight test. Note that in this simulation, it is assumed that an arbitrary polygonal area is given by the operator. Therefore, among various polygons introduced in Section 4.1, the hexagon described in Figure 8 was selected as the polygonal area for this simulation. MATLAB and gazebo cosimulation was performed in a ROS environment. To do this, we created a ROS master, and then the task assignment subsystem on MATLAB connected to the existing ROS mater. Therefore, the task assignment subsystem could exchange data with other ROS packages implementing other subsystems through publishers and subscribers. In addition, we used PX4 Software In The Loop (SITL) and gazebo to simulate the physical model of a quadrotor and to run PX4 firmware (v1.9.0) as the flight control software. By utilizing PX4 SITL and gazebo, we customized five quadrotors based on a 3DR IRIS drone, which can imitate the quadrotor dynamics accurately since it takes into account not only rigid body dynamics but also complex aerodynamic effects such as rotor-drags [26]. We also utilized MAVROS [27] as the interface between the path planning subsystem and the flight controller. The velocity command generated in the path planning subsystem was transferred to the flight controller at 40 Hz. Other parameter settings for this simulation are as follows. First, the mission altitude was set to 3 m, and the transition altitude of five UAVs was set to 5 m, 6 m, 7 m, 8 m, and 9 m, respectively. Second, the maximum flight speed was set to 3 m/s. Third, when the UAV enters a circle with a radius of 1 m from the target waypoint, we determined that the UAV has reached the target waypoint. Fourth, other parameters related to the task assignment subsystem were set to the same as in the previous simulations in Section 4.1.

**Figure 9:**
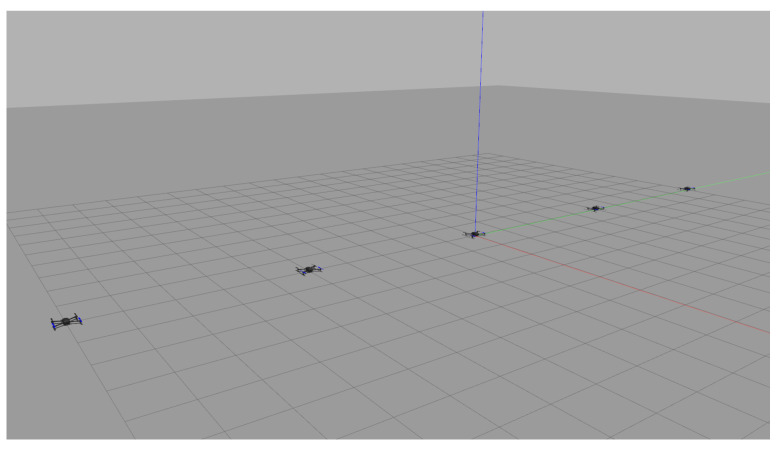
Simulation environment in Gazebo.

**Figure 10:**
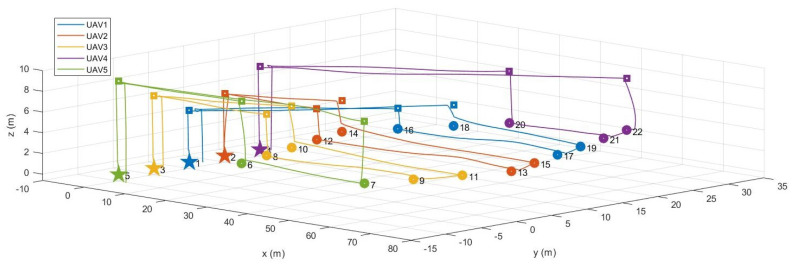
MATLAB and gazebo cosimulation results.

Figure 9 shows the gazebo simulation environment with five UAVs. As shown in Figure 9, five UAVs were placed at (0 m, 0 m), (0 m, 5 m), (0 m, −5 m), (0 m, 10 m), and (0 m, −10 m). Each UAV received the waypoint list from the task assignment subsystem, as shown in Figure 8d. For example, the waypoint list transferred to UAV1 was 1–18–19–17–16–1. The task management subsystem added several virtual waypoints for safe separation, as mentioned in Section 2.3. After the final waypoint list was generated, each UAV took off until it reaches the first waypoint and then moved in sequence to a series of waypoints. The coverage mission ended with all UAVs returning to their initial position after visiting all waypoints assigned to them. Figure 10 shows the overall flight process of five UAVs taking off from the ground and visiting the received waypoint list, then returning to the depot and landing. In Figure 10, the small squares depict the virtual waypoints added in the task management subsystem for safe separation. The solid lines are the flight trajectories traveled by the UAVs during the MATLAB and gazebo cosimulation. It can be seen from Figure 10 that thanks to using the proposed altitude separation, there were no collisions between members of the team during the mission.

## 5. Experiments

**Figure 11:**
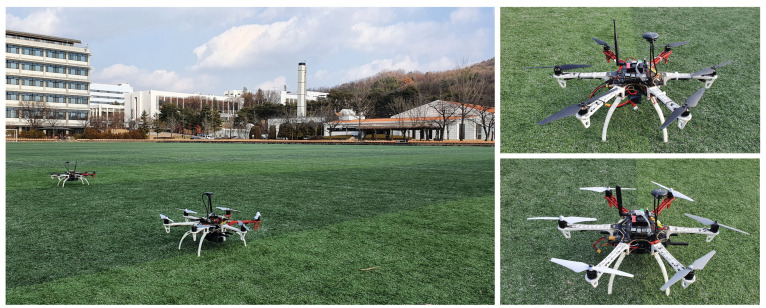
Outdoor experiment environment and quadrotor hardware platforms.

In this section, we present the outdoor experiment in which two hexacopters perform the coverage mission when the operator enters the mission through GCS. The most crucial parts of this outdoor experiment are the operator inputs through GCS, coverage mission for multiple areas, and real hardware implementation. The experiment was performed in an open space without any obstacles. Similar to the simulations performed in Section 4, we built two custom hexacopters with the DJI F550 frame, as shown in Figure 11. We used a Pixhawk4 running PX4 firmware (v1.9.0) as a Flight Control Computer (FCC). The FCC was responsible for calculating the motor inputs and controlling the hexacopter’s motion by estimating the states such as position, velocity, and attitude. Additionally, each hexacopter was equipped with an NVIDIA Xavier NX, as a companion onboard computer. The onboard computer was connected to the FCC to receive the list of waypoints, perform task management and path planning, and transfer the desired position, velocity, yaw, and yaw rate to the FCC. The laptop computer used as GCS hardware and two onboard computers mounted on hexacopters were connected to a single network over WiFi. All the hardware details are listed in Table 3.

**Figure 12:**
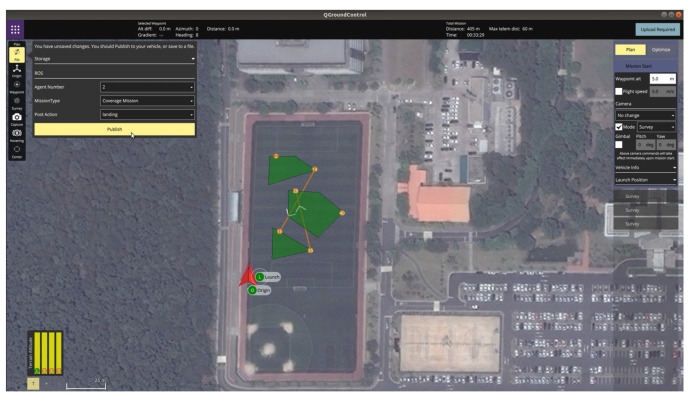
Mission plans through QGroundControl.

**Figure 13:**
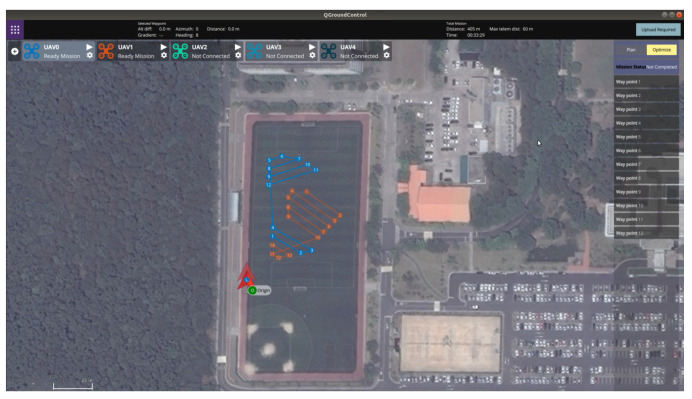
Optimization results shown in QGroundControl.

**Figure 14:**
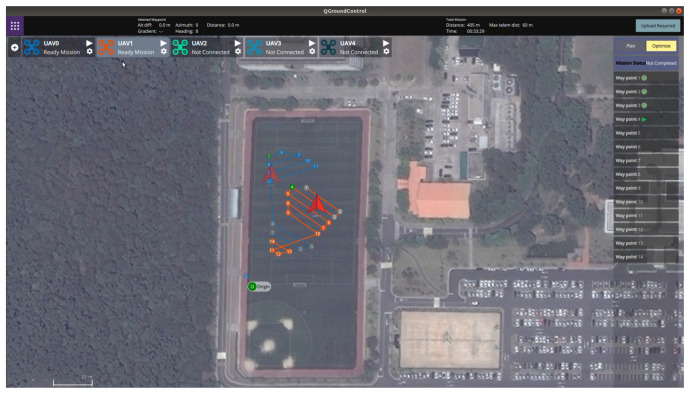
Waypoint status shown in QGroundControl.

Figure 12 shows the outdoor experiment scenario where the operator defined the coverage mission for three polygonal areas. As shown in Figure 12, the latitude and longitude of the origin of the inertial coordinate were set to 36.379720 deg and 127.364620 deg, respectively. The launch positions of two hexacopters were (36.379795 deg, 127.364639 deg) and (36.379852 deg, 127.364654 deg) in the GNSS coordinate, respectively. In addition, the spacing between waypoints was set to 5 m. Considering the GPS positional error, the mission altitude was set to 5 m, and the transition altitudes of two hexacopters were set to 8 m and 11 m, respectively. Figure 13 and Figure 14 show the visualization functions of GCS for the operator to monitor the mission. Figure 13 shows the optimization results received by the task assignment subsystem. It can be seen from Figure. Figure 13 that the back-and-forth movement could be generated in a direction parallel to the longest boundary line even if the operator did not enter the angle to generate the detailed waypoints within the polygon. Furthermore, the operator could identify the waypoint list assigned to each hexacopter before the flight starts and could confirm the progress of the mission. Figure 14 shows the status of each waypoint provided by the task management subsystem. As shown in Figure 14, three different colors were used to indicate the three types of the waypoint status; (1) gray waypoints that the hexacopter has already been reached, (2) green waypoints that the hexacopter is currently being followed (i.e., the target waypoint), and (3) default color waypoints that the hexacopter have not yet been reached. The waypoint status could also be checked with the green icons on the right panel, as shown in Figure 14.

**Figure 15:**
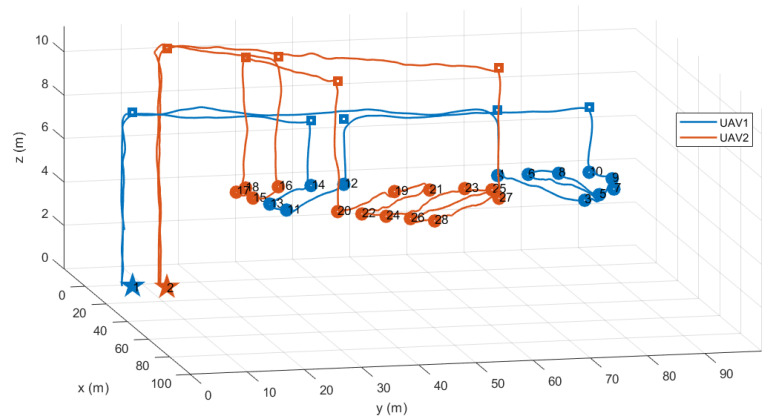
Flight trajectories.

Figure 15 shows the task assignment results and the flight trajectories of hexacopters recorded after the flight test was completed. As shown in Figure 15, 28 nodes were generated by the proposed graph building method to perform the coverage mission for three polygonal areas. Specifically, the waypoint lists assigned to the first and the second hexacopters were 1–14–13–11–12–10–9–7–8–6–5–3–4–1 and 2–27–28–26–25–23–24–22–21–19–20–18–17–15–16–2, respectively. Therefore, the travel distance of UAV1 and UAV2 calculated from the task assignment problem are 306.96 m and 306.43 m, respectively. Additionally, the objective function of the optimization problem was 613.65 m; the maximum flight distance was 306.96 m and the average flight distance was 306.69 m. The computation time required to solve the optimization problem was 4.61 s.

**Figure 16:**
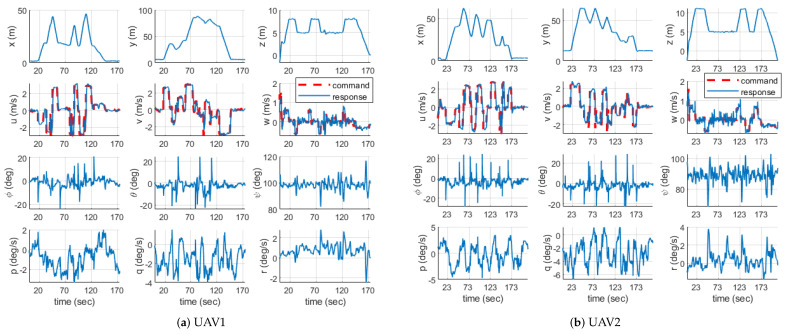
Time histories of states.

Figure 16 shows the time histories of each UAV’s states, including position, velocity, attitude, and angular rate recorded in the outdoor experiment. The total distance traveled by UAV1 and UAV2 were 337.64 m and 359.95 m, respectively. Since the virtual waypoints were added in the task management subsystem for safe separation, there was a slight difference between the distance the hexacopter actually traveled and the distance calculated in the task assignment subsystem. From these results, it can be concluded that the proposed system can perform area coverage missions more autonomously with multiple UAVs and is sufficiently applicable even in outdoor environments through real hardware implementation.

## 6. Conclusions

This study has attempted to establish a complete mission execution framework, from operator input to drone motor command for the autonomous and optimal area coverage mission using multiple Unmanned Aerial Vehicles (UAVs). In one communication network, the hardware system consisted of a laptop computer acting as a Ground Control Station (GCS) and several UAVs performing the mission. The software system was made up of five subsystems: (i) GCS to define the mission and monitor the whole progress of the mission, (ii) task assignment to allocate waypoints to each UAV, (iii) task management to check the waypoint status, (iv) path planning to generate a feasible path to the target waypoint, and (v) flight control to make a UAV fly to the desired path. In the task assignment subsystem, the proposed graph building method could lighten the computational complexity of the optimization problem determining the optimal pair between waypoints and UAVs while effectively generate the back-and-forth movement as well as prevent overlapping of flight areas between UAVs. The performance of the proposed system was verified through two simulations; one is through MATLAB simulation focusing on the validation of the task assignment subsystem and the other is MATLAB and Gazebo cosimulation for final validation before actual flight testing. Finally, outdoor experiments with real hardware implementations confirmed that multiple UAVs more autonomously cover multiple areas designated by the operator in the field.

For future work, this study will be expanded to following directions. First, in terms of task assignment, this study was limited in that all UAVs participating in the mission have the same capabilities. However, more research is needed to optimally distribute tasks between heterogeneous UAVs when each UAV has different maximum travel distances or different sensor specifications. Second, although this study did not consider cases where UAVs fail to reach the assigned waypoints, considerable work needs to be done to determine when mission replanning is necessary and how to resolve the replanning problem. Third, further research should be conducted on path planning strategies for carrying out the area coverage mission in a dynamic environment with obstacles previously unrecognized or moving obstacles. Lastly, although the UAV only visits the list of assigned waypoints in sequence in this study, future studies can be undertaken to perform detailed tasks at the waypoint (such as taking pictures or acquiring point cloud data). The final output of the area coverage mission will be 3D maps or 3D models that are increasingly available in agriculture, construction, mining, inspection, surveying, and public safety.

## Figures and Tables

**Table 1:** Task assignment results for the sample configuration.

	UAV1	UAV2	UAV3
Number of waypoints	4	4	8
Sequence of waypoints	1–8–9–11–10–1	2–6–7–5–4–2	3–18–19–17–16–14–15–13–12–3
Travel distance [m]	143.86	154.96	214.42
Avg. travel distance [m]	171.08		

**Table 2:** Task assignment results for various polygons.

		Triangle	Tetragon	Pentagon	Hexagon
Two UAVs	Max. distance [m]	180.31	260.69	238.12	260.38
	Avg. distance [m]	165.02	237.57	206.39	243.83
Three UAVs	Max. distance [m]	143.98	214.42	175.67	202.30
	Avg. distance [m]	142.43	171.08	160.13	182.37
Four UAVs	Max. distance [m]	140.76	149.73	153.75	170.59
	Avg. distance [m]	134.89	138.83	127.43	145.74
Five UAVs	Max. distance [m]	137.20	146.77	148.57	143.45
	Avg. distance [m]	130.67	137.16	125.96	133.29

**Table 3:** Hardware specification.

hexacopter platform	frame	DJI F550
	motor	DJI 2312E
	ESC	DJI 430 LITE
	propeller	DJI 9450
	battery	Lumenier 4S 5200 mAh
sensors	GPS	ublox M8N
computers	flight controller	Holybro Durandal (PX4)
	mission computer	NVIDIA Xavier NX

## Data Availability

Not applicable.
